# Patterns of Psychiatric Help Seeking Behaviour Among Patients With Psychotic Illness, Presenting at Mental Health Institute in Pakistan

**DOI:** 10.1192/bjo.2024.235

**Published:** 2024-08-01

**Authors:** Rajesh Kumar, Rahul Roy, Priyanka Devi

**Affiliations:** 1 LUMHS, Jamshoro, Pakistan; 2 SIR CJIP, Hyderabad, Pakistan; 3 West London NHS Foundation Trust, Crowthorne, United Kingdom; 4 PUMHSw, Nawabshah, Pakistan

## Abstract

**Aims:**

To identify the patterns of psychiatric help seeking behaviour among patients with psychotic illness presenting to mental health institute in Pakistan.

**Methods:**

This Descriptive, Cross-Sectional Design comprised of a sample of 103 patients with psychotic diseases, chosen via non-probability – consecutive sampling at Outpatient Department of Sir Cowasjee Jehangir Institute of Psychiatry, Hyderabad. The relatives of psychotic patients who were between 18 and 65 years of age with either gender and living with at least one family member were interviewed after taking informed written consent. An anonymous self-structured questionnaire containing inquiries pertaining to basic biodata, sociodemographic details, psychiatric diagnosis and disease particulars, pattern of help seeking and time and reasons for delay.

**Results:**

The mean age of the sample stood at 32 years (±9.5 SD). 1/3 of the sample comprised of male patients while only 24% were comprised of female population. The mean time elapsed after first episode psychosis till interview was 82 ± 32 months (7 years) while mean delay in help seeking to any helper was reported to be 41 ± 17 months (3.5 years). Majority of the patients approached first to faith healers (Aamil Baba, Witch Doctor, Pir, Religious Leader, Molvi, Imam or Religious Cleric) while only 1/5 of the patients approached to psychiatrists for treatment of first psychotic episode. The mean time duration to approach to psychiatrist after first episode psychosis was reported to be 73 ± 38 months (around 6 years).

**Conclusion:**

The study showed that most frequent source of health care for psychiatric patients were faith healers (Aamil Baba, Witch Doctor, Pir, Religious Leader, Molvi, Imam or Religious Cleric) as compared with one-third who went to qualified healthcare providers like psychiatrists or physicians. There is a huge delay in proper help seeking among psychiatric patients. Health education aiming at increasing awareness among general population regarding treatment options for psychiatric illness is recommended to improve the quality of life of people living in our locality.

